# Cooperative interactions between nano-antennas in a high-Q cavity for unidirectional light sources

**DOI:** 10.1038/s41377-019-0227-x

**Published:** 2019-12-11

**Authors:** Kévin G. Cognée, Hugo M. Doeleman, Philippe Lalanne, A. F. Koenderink

**Affiliations:** 1 Center for Nanophotonics, AMOLF, Science Park 104, 1098 XG Amsterdam, The Netherlands; 2 LP2N, Institut d’Optique Graduate School, CNRS, University of Bordeaux, 33400 Talence, France; 3 Van der Waals-Zeeman Institute, University of Amsterdam, Science Park 904, 1090 GL Amsterdam, The Netherlands

**Keywords:** Nanocavities, Nanophotonics and plasmonics, Single photons and quantum effects

## Abstract

We analyse the resonant mode structure and local density of states in high-Q hybrid plasmonic-photonic resonators composed of dielectric microdisks hybridized with pairs of plasmon antennas that are systematically swept in position through the cavity mode. On the one hand, this system is a classical realization of the cooperative resonant dipole–dipole interaction through a cavity mode, as is evident through predicted and measured resonance linewidths and shifts. At the same time, our work introduces the notion of ‘phased array’ antenna physics into plasmonic-photonic resonators. We predict that one may construct large local density of states (LDOS) enhancements exceeding those given by a single antenna, which are ‘chiral’ in the sense of correlating with the unidirectional injection of fluorescence into the cavity. We report an experiment probing the resonances of silicon nitride microdisks decorated with aluminium antenna dimers. Measurements directly confirm the predicted cooperative effects of the coupled dipole antennas as a function of the antenna spacing on the hybrid mode quality factors and resonance conditions.

## Optics: Next-generation optical sensors with unprecedented sensitivity

Scientists have developed a simple technique for enhancing the properties of hybrid plasmon-photonic resonators, opening the door to micro- and nanoscale optical technologies for use in vibrational spectroscopy, lasers, and solid-state lighting devices. Optical sensors with whispering gallery mode resonators, in which light waves travel around a concave surface, possess unprecedented sensitivity and have evolved into valuable tools for probing nonlinear optical phenomena and quantum electrodynamical principles. By investigating the hybridization of microcavities with multiple metal nanoparticles, Kévin Cognée and colleagues from AMOLF and the University of Amsterdam in the Netherlands, in collaboration with researchers from the University of Bordeaux in France, have developed plasmonic-photonic resonators composed of dielectric microdisks hybridized with pairs of plasmon antennas that increase light-particle interaction strength, tailoring their emission properties. The work holds the promise of next-generation analytical and diagnostic technologies.

## Introduction

Tailoring optical resonators to have any desired quality factor *Q* and mode volume *V* is a major endeavour in nano- and micro-optics as the basic stepping stone to controlling the light-matter interaction in diverse scenarios that range from cavity QED, to nonlinear optics, to vibrational spectroscopy, to building lasers and solid-state lighting devices^1,2^. Notably, it is desirable to independently control the field strength per photon (gauged by *V*), the resonator linewidth *Q*^3^, and the channel to which the resonator favourably couples with far-field radiation. For instance, when controlling the rate of spontaneous emission experienced by a quantum emitter placed in a resonator, it is desirable to control the Purcell factor $F = (3\lambda ^3/4\pi ^2)Q/V$ while at the same time tuning the cavity to the emitter frequency, making sure that the cavity linewidth is matched to the emitter spectrum^4,5^ and ensuring that light extraction occurs through one highly efficient channel. Similar arguments hold for strong coupling between light and matter^3,6,7^, SERS and cavity/molecular optomechanics^8^ and, generally, processes that at the same time need high field enhancement yet also matching of the linewidths to other experimental constraints. In the last decade, great progress has been made in realizing extremely confined resonators of $V \sim \lambda ^3/10^4$ and low *Q* ~ 20 on the one hand through plasmonics^5,7,9–11^ and high-Q microcavity resonators with $V \,>\, (\lambda /2)^3$ on the other hand^12^. Reaching very large *F* at intermediate $5\,< \,Q\, < \,10^4$ factors, however, has remained elusive, despite the large possible relevance for matching the linewidths of room-temperature emitters.

Recently, several groups have explored whether so-called hybrid plasmonic-photonic resonators could access the regime of deep subwavelength confinement, owing to their plasmonic constituents^4,13–16^, while at the same time inheriting larger quality factors from a dielectric microcavity character. Efforts in this direction include hybridizing single plasmonic nano-antennas with photonic resonances such as the whispering gallery mode (WGM) supported by Mie spheres, microtoroids, or microdisks or the localized modes in photonic crystal cavities^15,17–20^. Recent computational predictions indicate that hybrid modes offer Purcell factors exceeding those of the individual constituents, with *Q*-factors on the same order as those of the microcavity mode, and therefore with *V* profiting from the hybridization^4,14,16,21^.

**Fig. 1:**
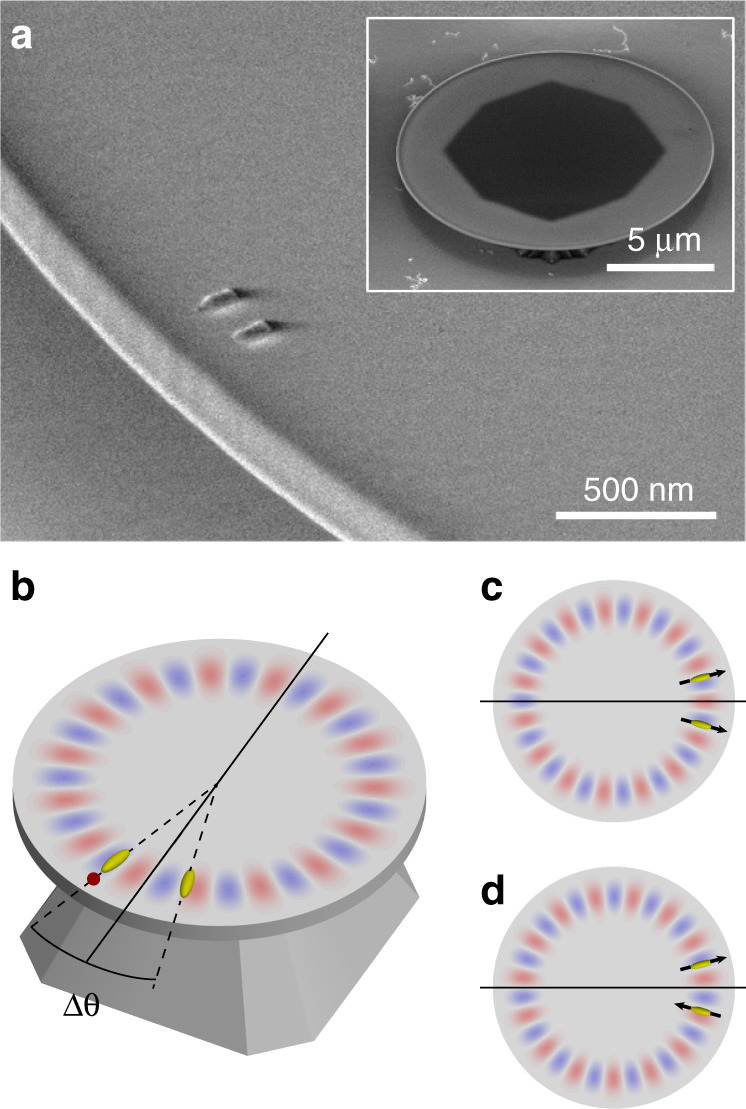
Hybrid plasmonic-photonic structure of an antenna-dimer on a microdisk. **a** Scanning electron micrograph (SEM, angled view) showing the geometry, consisting of two radially oriented aluminium nanorods on the perimeter of a silicon nitride microdisk. Inset: zoomed-out SEM of a full microdisk cavity. The dark angular pattern marks the contact with the silicon support pedestal. **b** Sketch of the geometry, where the antennas are separated by a subtended angle Δ*θ*. One antenna is driven by a spontaneous emitter (red dot). **c**, **d** Sketch of the symmetric and antisymmetric hybrid modes (mirror plane indicated as black line).

In this work, we consider the hybridization of microcavities with not one but multiple metal nanoparticles. This problem is interesting from three different perspectives. First, it is an implementation of cooperative scattering engineered by dipole–dipole coupling in a resonator, mirroring the physics of sub-radiant and super-radiant collective states in which many dipoles coupled to one cavity hybridize, thereby providing a classical precursor to the important quantum optics problem of cooperative emission^22,23^. Second, from an antenna point of view, it introduces the notion of phased array antennas into hybrid systems, with the associated control not only over Purcell enhancement but also over the distribution of light into far-field radiation channels^5,24^. The seminal example in free space is the so-called Yagi-Uda antenna, in which a single quantum emitter drives a single antenna element surrounded by a set of “director” scatterers to ensure unidirectional emission^25–27^. In this work, we present a minimal phased array on a WGM platform (Fig. 1) and show that this can similarly result in unidirectional emission. A third perspective instead focuses on the physics of the cavity modes in hybrids rather than the antenna physics. Indeed, our work is the first step of a plasmonic implementation of a proposition by Wiersig^28^, who proposed that dielectric scatterers on WGM cavities support chiral eigenmodes associated with exceptional point physics^29^. This work combines all three perspectives and explores the capabilities of plasmonic dimers to both enhance the hybrid emission and allow for directivity, here related to the circulation of light emitted into the cavity. Our theoretical analysis examines the distinct fingerprints in the mode lineshift and linewidths that may occur depending on the positioning of antennas in the WGM profile. At the same time, in vein of the proposition of Wiersig^28^ that eigenmodes can become chiral, we assess whether selective unidirectional emission in the case where a single antenna out of a pair is driven by an emitter is possible and with what directionality contrast and Purcell factor. This proposition can be seen as realizing a two-element directive phased array antenna design. We complement theory based on dipole-dipole interactions mediated by degenerate quasinormal modes (QNMs) with experiments, studying silicon nitride microdisk resonators coupled to dimers of aluminium nanorod antennas. We quantified the dependence of the perturbed mode frequencies and quality factors on antenna separation, finding direct evidence for cooperative antenna effects on the linewidth and lineshift that extend over large antenna separations.

This paper is structured as follows. First, we sketch an analytical model for describing *M* antennas coupled to a set of cavity modes. Next, we focus on the particular case of a WGM disk coupled to a plasmon dimer, examining the local density of states as well as the distribution of emission over clockwise and anticlockwise circulation in the cavity and in the far field. We then explain the spectral structure and the apparent unidirectional power distribution of emission by finding the QNMs (complex-frequency modes) of the coupled QNM equations. In the second half of this work, we report experiments, focusing on narrowband mode spectroscopy of WGM-antenna dimer hybrids.

## Results

### Model

The starting point of our work is to consider *M* antennas as *M* polarizable objects with dipole moments ${\mathbf{p}}_i$ ($i = 1 \ldots M$) and to model their mutual interaction through the master microdisk cavity with a quasinormal-mode formalism. Previous related works include, on one hand, coupled-mode theory and Green’s function theory for polarizable objects coupled to resonators^4,30^ and, on the other hand, works using QNMs for a semi-analytical model of the optical properties of a plasmonic resonator interacting with a single quantum object^31^, as well as with ensembles of classical dipolar oscillators^23,32^. The present model features two extensions. First, it addresses the important case of degeneracy of the underlying bare resonator modes, and second, it considers changes in the resonator properties induced by the polarizable objects. This aspect relates to the perturbation theory of resonators^20,33,34^. Both features are essential for the properties reported hereafter.

In brief, the dipole moments induced in a set of antennas^23,35^, each of polarizability ***α***(*ω*) and positioned at **r**_*i*_ on a microdisk cavity, are approximately given by

1 $${\mathbf{p}}_i = {\it{\epsilon }}_0\boldsymbol{\alpha} (\omega )\,\times\,\left[ {{\mathbf{E}}_{{\mathrm{dr}},{\mathrm{i}}} + {\sum \limits_{k = s,as}} a_{k}{\tilde{\mathbf E}}_{k}\left( {{\mathbf{r}}_i} \right) + \mu _0\omega ^{2}{\sum \limits_{j = 1}^M} {\mathbf{G}}_0({\mathbf{r}}_i,{\mathbf{r}}_j,\omega ){\mathbf{p}}_j} \right]$$

where ${\mathbf{E}}_{{\mathrm{dr}},{\mathrm{i}}}({\mathbf{r}}_i,\omega )$ represents the externally applied driving field at antenna *i*, while the other two terms quantify the dipole-dipole interactions between antennas. These are separated into interactions via the cavity QNMs (${\tilde{\mathbf E}}_k\left( {{\mathbf{r}}_i} \right)$, second term) and interactions via all other modes of the system, lumped into the background Green function **G**_0_. The formalism by which cavity QNMs are singled out to describe a resonator from a full-system Green function is presented in depth in the “Methods” section and builds on refs. ^36^^–38^. In this work, we focus on a pair of degenerate QNMs typical of WGM cavities, i.e., a pair of standing modes ${\tilde{\mathbf E}}_s$ and ${\tilde{\mathbf E}}_{as}$, which vary as cos(*mθ*) resp. sin(*mθ*) in the azimuthal direction. These modes can be equivalently regrouped by linear combination as clockwise and anticlockwise running modes. In this work, we focus on the physics of a single emitter placed in this system at location **r**_dr_ and modelled as a drive dipole **p**_dr_. As proven in the Methods section, the excitation coefficients of the two QNMs are self-consistently determined by the multiple scattering interactions between antennas through the cavity modes that are directly fed by the emitter as

2 $$a_{s,as} = \frac{{ - \tilde \omega }}{{\omega - \tilde \omega }}\left[{\tilde{\mathbf E}}_{s,as}({\mathbf{r}}_{{\mathrm{dr}}}) \cdot {\mathbf{p}}_{{\mathrm{dr}}} + \mathop {\sum }\limits_{i = 1}^M {\tilde{\mathbf E}}_{s,as}({\mathbf{r}}_i) \cdot {\mathbf{p}}_i\right]$$

We evaluate the Purcell factor that the drive dipole experiences and the directionality of emission into the cavity, determined from the (anti)clockwise mode amplitudes $a_{cw/ccw}(\omega ) = \frac{1}{{\sqrt 2 }}(a_s(\omega ) \mp ia_{as}(\omega ))$ as parameter $\sigma = \frac{{|a_{cw}|^2 \,-\, |a_{ccw}|^2}}{{|a_{cw}|^2 \,+\, |a_{ccw}|^2}} = \frac{{|a_s \,-\, ia_{as}|^2 \,-\, |a_s \,+\, ia_{as}|^2}}{{|a_s \,-\, ia_{as}|^2 \,+\, |a_s \,+\, ia_{as}|^2}}$. This directivity equals (−)1 if all light in the cavity is circulating in the (anti)clockwise cavity mode or 0 if light is distributed equally over both circulation directions.

### LDOS and directionality

**Fig. 2:**
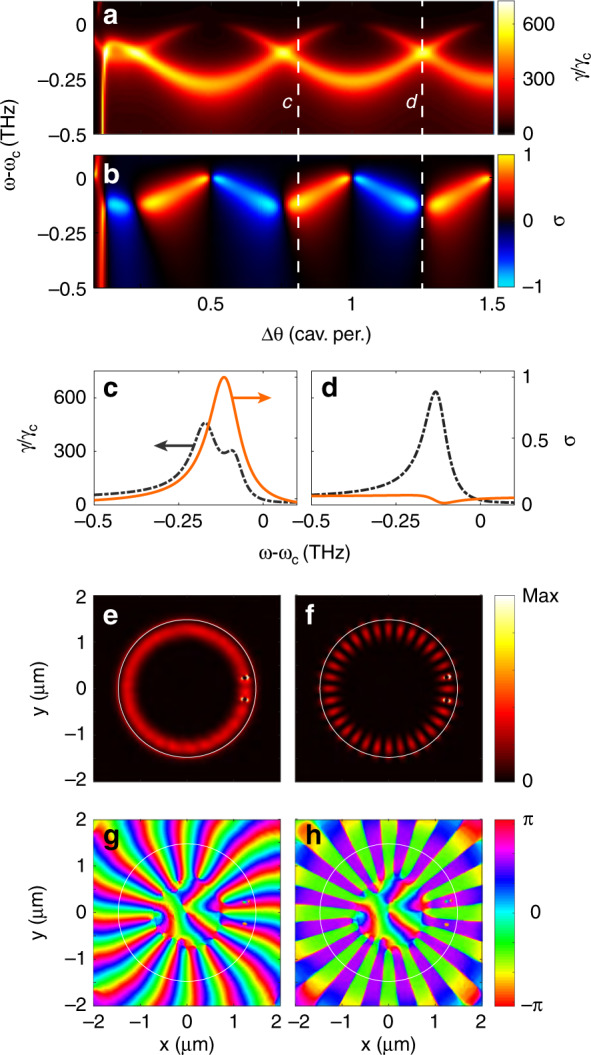
Predicted emission enhancement and directivity for antenna-dimer microdisk hybrids. **a** Local density of states enhancement for a source at antenna 1 as a function of the antenna separation expressed in the cavity mode profile azimuthal period and as a function of the frequency. The bare cavity resonance *ω*_*c*_ is at 360 THz. **b** Directionality of the emission into the cavity. **c**, **d** LDOS enhancement (black dashed) and directionality (orange solid) versus frequency for two antenna separations, as indicated by vertical dashed lines in (**a**, **b**), just off degeneracy (**c**) at unit directionality and exactly on degeneracy (**d**) with no directionality. **e**, **f** Simulated intensity and **g**, **h** phase of the radial component *E*_*r*_ of the electric field on the top interface of the microdisk cavity dressed by two aluminium antennas. The antenna separation is 0.94 cavity period for (**e**, **g**) and 1 cavity period for (**f**, **h**), and the simulation is driven by a radial dipole placed 10 nm radially outward from the top antenna. In (**e** and **g**), the intensity is almost perfectly homogenous in the azimuthal direction, with the phase indicating clockwise propagation. In contrast, (**f** and **h**) exhibit a standing wave pattern, as antennas and dipoles couple only the symmetric cavity mode.

While our model does not require that, e.g., antennas are identical or symmetrically placed, we focus on plasmon dimers (*M* = 2) of antennas that are identical, both radially oriented and placed at the same distance from the rim of a microdisk cavity (Fig. 1b) with *Q* = 10^4^ and deliver a Purcell enhancement of 75 above the microdisk and in the plane of the antennas. We parametrize the distance between antennas with their angular separation Δ*θ*. For the cavity, we take as azimuthal mode number *m* = 22 and *ω*_*c*_ = 360 THz as the cavity resonance frequency, implying operation near 800 nm, near the wavelength of the experiments also reported in this work. These numbers are typical for silicon nitride microdisks in the near infrared of ~4 μm in diameter and are consistent with those of ref. ^4^. The radially oriented antennas couple to a TE mode, with plasmon antenna parameters commensurate with those of Au plasmonic dipolar antennas, retrieved from full-wave simulations for gold nano-ellipsoids (see “Methods”). We place the source at a distance of 60 nm from the centre of one of the two antennas and assume that the dipole is polarized along the disk axis (see Fig. 1). In the absence of the microdisk, the Purcell factor provided by the coupled nanorod is ≈200 at antenna resonance. Figure 2 shows the local density of states normalized to that in a vacuum as a function of the frequency around the bare cavity resonance and as a function of the angular separation between the antennas, as calculated using the simple formalism that we presented. For reference, if only a single antenna is present, the hybrid antenna-cavity construct presents a Fano lineshape in the local density of optical states (LDOS), with a peak LDOS enhancement of almost 700, as was also verified independently of the approximations of the model by full-wave simulations^4^. This value is almost an order of magnitude larger than the one provided by the bare cavity alone and more than three times higher than the *maximum* LDOS enhancement of 200 provided by only a bare antenna at its resonance. Figure 2a reveals that in the dimer case, the LDOS enhancement reaches similar large values but with two resonant features that present a distinct oscillatory behaviour as a function of the angular separation between the antennas. Additionally, in the presence of two antennas, the hybrid modes can still be classified by symmetry, as there is mirror symmetry through the line from the cavity centre to the midpoint between the antennas (cf. Fig. 1). Thus, one of the QNMs is symmetric and displays a node in the radial field between the antennas while having both antennas polarized in the same radial direction, while the other QNM is antisymmetric, with a null between the antennas, and both antennas are polarized in opposite directions. When the antennas are shifted by a multiple of half a cavity-mode-profile azimuthal period, one QNM is not perturbed by the antennas, while the other is perturbed by both, giving a large broadening and frequency shift. Midway between these conditions, both modes have identical overlap with the antennas, meaning they are shifted equally in real frequency and in Q, maintaining degeneracy. In the first scenario, an emitter placed at one of the antennas couples only to the symmetric mode, which is maximally perturbed. In the second scenario, both modes contribute to the LDOS, explaining why the degeneracy point also corresponds to the highest LDOS. This LDOS is approximately the same in value as in the single-antenna hybrid: while *two* hybrid modes contribute to the LDOS instead of one, each of them contributes only half as much, owing to the fact that the antennas are more weakly coupled to the pertinent cavity modes than in the single antenna case by virtue of not being at the mode maxima of either the S or AS mode.

The picture sketched above essentially repeats itself with increasing antenna separation, with a repetition period equal to Δ*θ* = *π*/*m*, reflecting the fact that the antenna interaction is dominated by the cos(*mθ*) resp. sin(*mθ*) dependence of the cavity modes. At very short distances, however, the behaviour is qualitatively different, with a very strong perturbation (frequency shift and broadening) of the cavity modes. The physical picture is that at close distances (below, say, *λ*/2*π*), the two antennas hybridize to form a symmetric, bright, strongly polarizable bonding combination that is blueshifted and an antisymmetric, dark, weakly polarizable anti-bonding mode that is redshifted from the bare antenna resonance. This behaviour is approximately captured in our model through **G**_0_ in Eq. (1), i.e., through the Green function of the system without cavity modes, taken to first approximation as the free space Green function, which obviously contains near-field 1/*r*^3^ and mid-field terms. As explained in the Methods section, an exact quantitative treatment of this regime would require accounting for terms cut out of our QNM expansion by limiting it to just two cavity modes. The hybridization physics with the cavity modes is rich since when the two antennas hybridize, at a certain distance, the redshifting dark dimer mode tunes through resonance with the cavity, which had its bare resonance chosen to the red of the single antenna resonance. At this distance, the dark mode very strongly perturbs the cavity. On the other hand, the coupling strength of the blueshifting bonding mode becomes weaker with reduced particle separation, as blueshifting tunes it out of the cavity resonance.

For the case of an emitter driving a single nano-antenna, in the absence of a second antenna, symmetry dictates that emission will be injected into only the symmetric mode, i.e., with equal contributions of the clockwise and anticlockwise directions. The presence of the second antenna, however, implies that both the symmetric and antisymmetric QNM can contribute to the LDOS. Hence, it is interesting to resolve in which direction the light is emitted into the cavity. We find that the second antenna can make the emission largely unidirectional. Figure 2b represents the splitting ratio σ. Essentially unidirectional emission is achievable at combinations of the frequency and geometry that are close to, but not at, the mode degeneracy points ${\mathrm{\Delta }}\theta = (n + \frac{1}{2})\pi /m$ and that bring simultaneously large LDOS enhancement. Figure 2c, d highlights the behaviour for two distinct antenna separations, namely, Δ*θ* = 0.81 cavity periods (i.e., just beyond the degeneracy at 0.75) and, right at degeneracy, Δ*θ* = 1.25 cavity periods. Unidirectional emission *σ* = 1 can coincide with large LDOS enhancement, exceeding a factor of 300. This situation occurs at angular separations close to, but not at, a point of maximum LDOS. Conversely, near-equal power splitting at near maximum LDOS enhancement (enhancement >600) occurs at points of degeneracy. It should be noted that while we report the total LDOS in this work (including also nonradiative enhancement), ref. ^4^ for single antennas shows that up to 95% of the radiated power can be extractable through the cavity loss channels. The implication is that for the geometry in Fig. 2c, if the microdisk would be addressed by a tapered waveguide as the main input/output channel, the fluorescence could be efficiently captured into just one waveguide direction. Conversely, by reciprocity, one would expect the emitter to be addressable from just one waveguide direction. The basic requirements for this behaviour in emission and excitation are (1) the correct separation between the antennas and (2) placement of the emitter such that it dominantly couples to just one of the antennas instead of coupling to both antennas equally (emitter between the two antennas), dominantly coupling to just the (spoiled) cavity (emitter on the disk perimeter but more than 50 nm away from the surface of any antenna), or dominantly coupling to free space (emitter well away from the antennas and the WGM profile). This means our predictions hold as long as the emitter is placed within the near-field hot spot of the intended feed antenna, i.e., within 20 nm or so off the distal end for a nanorod antenna realization. On basis of ref. ^39^, we expect the predictions to also hold if the feed antenna to which the emitting dipole couples is replaced by a dimer gap antenna of similar dipole polarizability, where placement of the emitter in the gap could significantly enhance the LDOS. Finally, we note that while *M* = 2 antennas are already very successful in creating unidirectionality, our model is easily extended to more than two antennas. For creating unidirectional emission, we note that similar performance is possible for *M* = 3 antennas, while generally, at larger *M*, there is no further improvement in unidirectionality but a large penalty in the LDOS. This is a consequence of the reduction in Q with the addition of antennas.

To verify that the semi-analytical predictions from our model are not an artefact of the approximations, we performed full-wave simulations to verify the occurrence of unidirectional emission (see ref. ^4^ for LDOS benchmarking). We analysed a Si_3_N_4_ microdisk (thickness of 200 nm, diameter of 2.95 μm) decorated with two aluminium nanorod antennas (100 nm long, 50 nm high, and 40 nm wide). We first evaluated the bare cavity mode profile (*m* = 16 mode at 396.675 THz, *Q* = 4000) and its frequency shift upon perturbation by a single antenna (at 396.3 THz, *Q* = 800). From these, we predicted the operation points (frequency and antenna spacing) for unidirectional and completely symmetric emission to occur at antenna separations of 0.94 resp. 1 cavity period, both at a frequency of 396.650 THz. Next, for these operation conditions, we performed driven simulations with a single drive dipole next to one antenna. Figure 2e–h shows the cycle-averaged field intensity |*E*|^2^ and the phase for both cases. In the first case, we find the signature of constant field intensity and circulating phase corresponding to the excitation of a travelling wave, while in the second case, we find the constant-phase field intensity nodes and antinodes characteristic of a standing wave. The slight residual fringe contrast for the unidirectional case indicates that over 96% of the energy travels in a single direction. Thus, the simulation confirms the predicted phased array action, as well as the operation points at which the distinct scenarios occur.

### Complex-frequency analysis

**Fig. 3:**
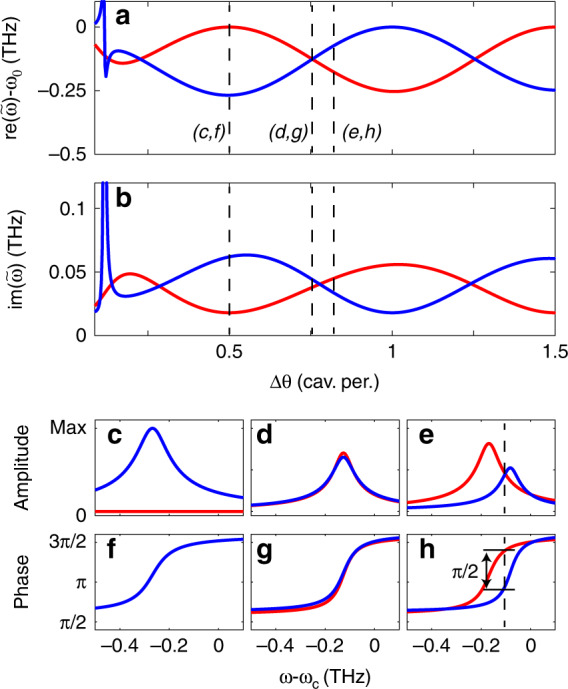
Complex eigenfrequencies for the antenna-dimer microdisk hybrid system. Real (**a**) and imaginary (**b**) eigenfrequency of the symmetric (blue) and antisymmetric (red) hybrid QNMs versus the angular separation between antennas. The amplitude (**c**, **d**, **e**) and phase (**f**, **g**, **h**) show the physics underlying directional emission at three salient antenna separations (dashed vertical lines in **a**, **b**). Since the amplitude of the symmetric mode is null in **c**, its phase is not defined and therefore not plotted in (**f**). The dashed line in (**e**, **h**) is a guide for the eye. Where one mode is maximally perturbed and the other is not (**c**, **f)**, the source excites only one of the two hybrid WGMs (symmetric, blue curves). Where the symmetric and antisymmetric modes are perturbed equally (**d**, **g**), both modes are excited equally and in phase, leading to no directionality. Just away from degeneracy (**e**, **h**), one can achieve equal amplitude and a *π*/2 phase difference, leading to unidirectionality.

The spectral structure, i.e., the Fano lineshapes, and the unidirectionality evident from Fig. 2 clearly involve the interference of several modes. This structure can be further understood from a complex-frequency eigenmode analysis of the coupled antenna-cavity equations. One can view Eq. (1) in the absence of the driving term (setting **E**_dr,i_ = 0) as a linear system **Ax** = 0 for the excitation coefficients ${\mathbf{x}} = [{\mathbf{p}}_i,a_k]$ of antennas and cavity modes. This equation defines complex-valued dressed eigenfrequencies $\tilde{\omega}^\prime$ through the condition ${\mathrm{det}}{\mathbf{A}}(\tilde \omega \prime ) = 0$. These represent the complex eigenfrequencies of the hybrid system QNMs. Figure 3a, b represents the real and imaginary parts of the eigenfrequencies. This analysis confirms the oscillatory behaviour of both the real and imaginary parts of the frequency with the angular antenna separation. Notably, at points where the antenna separation fits the distance between cavity mode antinodes, one of the two cavity modes is neither shifted in Q nor in frequency from the bare mode. In this configuration, the other mode is maximally shifted in both Q and frequency. At points of degeneracy in the real part of the frequency, the QNMs also have identical Q, with both experiencing approximately half the shift that is seen at points of maximum mode separation.

Considering the symmetry of our system and the fact that we expect two perturbed solutions close to the unperturbed cavity (complex) frequency $\tilde \omega _c$, the frequency shift of the two QNMs with respect to $\tilde \omega _c$ can be effectively parametrized through^28,33^

3 $$\frac{{\tilde \omega _{s,as\prime } - \tilde \omega _c}}{{\tilde \omega _c}} = \frac{{ - {\it{\epsilon }}_0\alpha \left( {\tilde \omega _c} \right)\tilde E_r^2\left( {r_i,z_i} \right)\left[ {1 \pm \cos \left( {m{\mathrm{\Delta }}\theta } \right)} \right]}}{{1 \pm \mu _0\tilde \omega _c^2\alpha \left( {\tilde \omega _c} \right)g_0\left( {\tilde \omega _c} \right)}}$$

where *i* designates equivalently 1 or 2, $\tilde E_r^{}$ is the radial component of the WGM QNM and ±cos(*m*Δ*θ*) accounts for the coherent addition of the two perturbers, which depends on their relative position in the mode profile of the S and AS mode. The denominator accounts for near- and mid-field hybridization correction effects on polarizability that are mediated by the background Green function $g_0(\tilde \omega _c) = {\mathbf{u}}_{r_i} \cdot {\mathbf{G}}_0({\mathbf{r}}_i,{\mathbf{r}}_j,\tilde \omega _c){\mathbf{u}}_{r_j},i \,\ne\, j$ from antenna to antenna. For sufficiently large antenna separation, the denominator of Eq. (3) is essentially equal to one, and the complex detuning ${\mathrm{\Delta }}\tilde \omega = \tilde \omega _{as}\prime - \tilde \omega _s\prime$ between the modes S and AS traces

4 $$\frac{{{\mathrm{\Delta }}\tilde \omega }}{{\tilde \omega _c}} = \frac{{\tilde \omega _{as}\prime - \tilde \omega _s\prime }}{{\tilde \omega _c}} = \frac{\alpha }{{\tilde V}}{\mathrm{cos}}(m{\mathrm{\Delta }}\theta )$$

here, $\tilde V(r_i,z_i)$ is the effective complex mode volume^38^ of each WGM of the unperturbed cavity at the radial position of the antenna [without the trivial cos(*mθ*) dependence, therefore being identical for the S and AS modes]. Aside from the expected inverse dependence of shift on the mode volume, this result also highlights the role of the phase of the polarizability. Since the complex mode volume is almost real in our example, the phase of the polarizability directly sets the balance between the real and imaginary part of the frequency shift. If the antennas are red (blue) detuned compared to the cavity, the frequency splitting between the symmetric and antisymmetric mode is (anti-)correlated with the difference in linewidth. If the antennas are on resonance with the cavity, there is no frequency splitting, but instead, the amplitude difference in linewidth is maximal. In other words, in that limit, the cavity is near degeneracy, but the two modes have very different Q values. The example considered in Figs. 2 and 3 corresponds to blue detuning (mainly a frequency shift).

Unidirectionality and LDOS enhancement can now be understood from the amplitude and phase with which the two QNMs are driven by a point emitter, calculated from the overlap (inner product) between the eigenvectors of **A** and the driving from a single dipole source. Directionality of emission occurs as a consequence of interference of the symmetric and asymmetric modes, with strict unidirectionality requiring destructive interference in one direction. Thus, essentially, the first requirement for perfect unidirectional emission to occur is that the localized excitation at one of the antennas must have the same projection on the hybrid basis. The second condition is that the relative phase is appropriate for destructive interference in the clockwise (anticlockwise) direction (with simultaneous constructive interference in the other channel guaranteed by symmetry). Figure 3c–h reports the amplitude and phase of excitation of the two modes for three distinct antenna separations for the example system considered in Fig. 2. In the first case (for an antenna distance Δ*θ* = 0.5), the antenna separation fits the distance between mode antinodes, meaning that the distance is half-integer in units of the cavity period. The dipole emitter couples only to the strongly perturbed symmetric normal mode of the system, so there is no directionality. Next, we consider an antenna separation chosen right at degeneracy (example chosen is Δ*θ* = 0.75). Again, the emission is equally distributed over both directions. The mechanism is, however, different from that at work at half-integer antenna distances. Indeed, now both modes of the system are excited instead of just a single one, but as the excitation has equal phase for both, there is no constructive/destructive interference. For a separation just away from degeneracy (example chosen is an antenna distance Δ*θ* = 0.81 cavity periods), the emitter can still couple to both modes of the system but with a phase difference. Indeed, by appropriately choosing the frequency, one can obtain a *π*/2 phase difference at equal excitation amplitude for the two modes, leading to perfect unidirectionality. The sign of unidirectionality is controlled by choosing Δ*θ* on opposing sides of the degeneracy point.

We finally note that this mechanism for unidirectional emission is distinct from the interesting exceptional point studies reported by Peng et al. in ref. ^29^, obtained by perturbing a WGM cavity with two near-field probes as scatterers. In a true exceptional point scenario, unidirectionality is intrinsic to the eigenmodes and not due to phase relations in the linear superposition of modes, as upon coalescence of the eigenfrequencies, the remaining eigenmode is chiral. In contrast, here, we exploit the asymmetric location of the emitter at just one antenna for unidirectionality, while our eigenmode set still retains even and odd parities. The mechanism relies on tuning the operation point near, but not on, mode degeneracy. An exceptional point instead requires the geometry to break parity symmetry by either using different radial positions, considering two geometrically different antennas, or adding a third antenna^40^. While outside the scope of this work, our QNM-based model for *M* antennas at arbitrary cavity locations (“Methods” section) does provide a comprehensive framework for analysing and designing these exceptional point optical cavity systems. The model quantitatively accounts for multiple scattering and antenna-antenna interactions, and the exceptional point physics is revealed from an optical mode analysis instead of requiring postulation by a non-Hermitian Hamiltonian parametrization, as is common in the literature^29^.

### Spectroscopy of hybrid microdisk devices

**Fig. 4:**
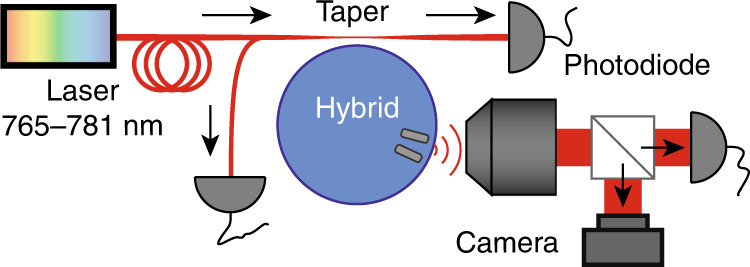
Setup schematic, indicating tapered-fibre excitation of microdisks with a narrowband tuneable diode laser with simultaneous recording of the taper reflection, taper transmission and out-of-plane scattering collected with an objective onto a camera or photodiode.

We report experiments that interrogate the cavity mode perturbation physics, i.e., the predicted shift in frequency and change in linewidth of the modes in Eq. (4), depending on the complex polarizability, the mode volume, and the azimuthal mode number. To this end, we apply tapered-fibre-mode spectroscopy to samples based on Si_3_N_4_ disks, hybridized with aluminium antennas (see “Methods” section). The experiments are performed on microdisks that are 15 μm in diameter and 200 nm in thickness with two ≈130 nm long, ≈50 nm wide and ≈40 nm thick radially oriented Al antennas, placed 300 nm from the disk edge (Fig. 1a). The disks stand on a ridge that is 150 μm in width and height, such that they are accessible for optical fibre taper coupling. A tapered optical fibre setup (Fig. 4, Methods section) provides excitation by an external-cavity diode laser that is widely tuneable yet narrowband (New Focus Velocity) at approximately 780 nm. The observables that we can simultaneously collect are fibre taper transmission, fibre taper reflection and out-of-plane scattering, collected with a microscopy setup. The microscope allows real space and Fourier space imaging of the scattering. We interrogated 70 cavities corresponding to a duplicated set of 35 different hybrid configurations where the separation angle between antenna varies, by design, from 0.8 to 13.5° (0.1 to 1.8 μm), i.e., ≈0.2 to 3.45 cavity azimuthal periods of the mode profile for our QNMs of interest (azimuthal mode numbers of 80 < *m* < 86 fall within our scan range).

**Fig. 5:**
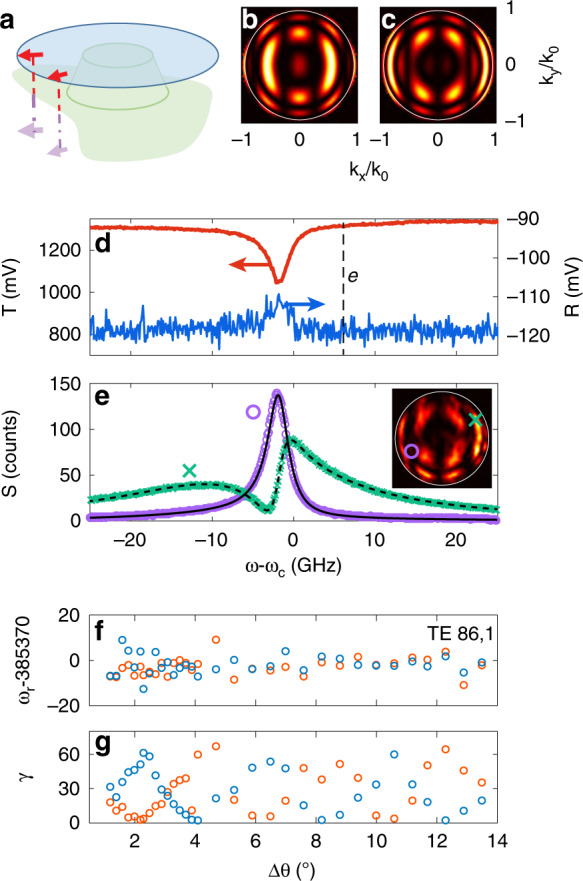
Scattering properties and eigenfrequencies of antenna-dimer microdisk hybrids. **a** Sketch of image dipole analysis explaining the far-field radiation pattern of the symmetric (S) QNM. **b**, **c** Predicted approximate angular radiation patterns into the air side for S and AS QNMs, plotting intensity per solid angle as a function of in-plane momentum in units of *ω*/*c*. **d** Taper transmission (red) and reflection (blue) diode signals for an antenna dimer (separation Δ*θ* *=* 11.2° (1.46 μm)) on a microdisk versus frequency relative to *ω*_ref_ = 385.37 THz. **e** Inset: collected radiation pattern at frequency labelled **e** in panel **d**. Purple and green curves mark the °,×-collected scattered intensity at chosen (*k*_*x*_,*k*_*y*_) indicated in the inset versus the driving frequency. **f** Resonance frequencies and **g** linewidths in GHz of the symmetric (blue) and antisymmetric (red) modes as a function of the antenna separation for antennas hybridized with the 1st radial mode at azimuthal mode number *m* = 86 (TE 86–1). The largest variation is in the linewidth (panel **g**). The resonance frequencies show a spread due to disk fabrication variations. These cancel out when considering *differences* in frequency.

Figure 5 shows a typical raw data set measured on a single device (antenna separation Δ*θ* = 11.2° (1.46 μm) for *m* = 80, second radial order). The transmission spectrum through the tapered fibre clearly shows power transfer to the cavity, with the lineshapes of two Lorentzian minima commensurate with a broad and narrow QNM. Depending on the geometry, these are not always clearly separable, particularly since the coupling strength to tapered-fibre channels depends strongly on the mode linewidth or when modes are very close to degeneracy. The reflection signal generally shows significant reflection features coincident with the transmission signature, however with asymmetric lineshapes that suggest interference with parasitic contributions (e.g., parasitic reflections at fibre connections). Finally, the scattering signal shows very clear Fano features, indicative of the coherent addition of the radiation patterns of the symmetric and antisymmetric QNM. Qualitatively, these radiation patterns can be understood both for the symmetric and for the antisymmetric QNMs as the sum of interfering dipole contributions (sketch in Fig. 5a (bright QNM only), predictions in Fig. 5b, c). These predictions are formed as the coherent sum of the symmetric resp. antisymmetric dipole combinations (dipoles located at the antenna centres, radially oriented). This leads to interference fringes in the far-field radiation pattern of both the S and AS modes. Notably, for small Δ*θ*, the dipoles are almost parallel, and the field radiated by the antisymmetric AS mode exhibits a dark fringe centred around *k*_*x*_ = 0. For each dipole, one further needs to account for the reflective air-silicon interface above which it is located, as highlighted in the sketch (Fig. 5a). The interface effect gives rise to an additional circular fringing concentric with *k*_*x*_ = *k*_*y*_ = 0 (vertical emission). An example measurement of a radiation pattern is shown in Fig. 5e (inset), which excellently agrees with the prediction for the asymmetric QNM. Scattering spectra at a select set of wave vectors chosen at salient features in the radiation pattern directly reveal the coherent superposition of QNMs through Fano lineshapes, as shown by the representative curves in Fig. 5e for the wave vectors marked in the inset. The advantage of these scattering spectra is that they are essentially background free, as the cavity excitation is through the taper, not through free space.

We extract QNM frequencies and Q values from the raw data by simultaneous Fano fits to transmission, reflection and scattering (data plotted in Fig. 5d, fit approach, see “Methods”). Figure 5f, g presents the dependence of the frequency and quality factor that results from fitting data on many hybrid devices with varying antenna-antenna spacing, yet for the cavity mode (TE, *m* = 86, and first radial order antenna, estimated from finite element simulations for a 15 μm diameter Si_3_N_4_ microdisk) and antenna size also considered in Fig. 5d, e. The oscillatory behaviour of the perturbed frequencies of the symmetric and antisymmetric QNM with antenna separation is especially clear in the linewidth, where the symmetric and antisymmetric QNM show anti-correlated behaviours. The real frequencies show much smaller variations, which are further masked by frequency variations between devices that arise from small fabrication inaccuracies. Indeed, spectroscopy on devices without antennas shows that the bare cavity frequencies themselves vary by ~100 GHz or, equivalently, by approximately 0.2 nm in wavelength, equating to a spread in disk diameters of approximately 3.5 nm.

**Fig. 6:**
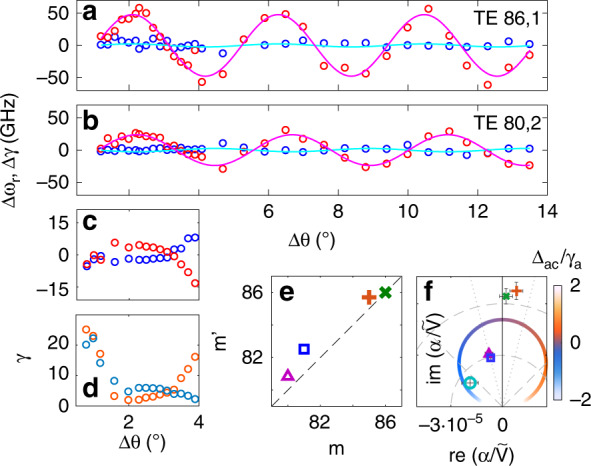
Mode splitting versus antenna spacing for dimer antennas hybridized with microdisk modes of various azimuthal and radial quantum number. **a**, **b** The *difference* in resonance frequency (blue, weakly varying) and linewidth (red, strongly varying) between symmetric and antisymmetric modes as a function of the antenna separation for antennas hybridized with **a** the 1st radial mode at azimuthal mode number *m* = 86 (TE 86–1) and **b** a mode of larger mode volume and different azimuthal mode number (2nd-order radial mode *m* = 80, TE 80–2). **c**, **d** show a similar study with strongly blue-detuned antennas, where **c** shows the difference in resonance frequency (blue) and linewidth (red) and **d** reports the individual linewidths of symmetric (blue) and antisymmetric (red) modes. **e** Azimuthal mode number *m*′ extracted from fitting the oscillation in the perturbed frequency to Eq. (10) versus the simulated mode number *m* for all WGM modes in the laser bandwidth. **f** Polar representation of the measured complex-valued $\frac{\alpha }{{\tilde V}}$ obtained by fitting the amplitude and phase of oscillation in frequency and linewidth to Eq. (10) [Δ: TE 80–2, □: TE 81–2, +: TE 85–1, ×: TE 86–1 for the on-resonant antenna and ◯ for the strongly blue-detuned antenna]. For reference, the circular curve shows the expected frequency dependence of $\frac{\alpha }{{\tilde V}}$ for a Lorentzian polarizability [choosing $\tilde V = 300\lambda ^3$ and an on-resonance extinction cross section of 0.12 μm^2^]. With frequency detuning Δ_ac_ from antenna resonance (colour coding of the curve, in units of antenna linewidth), the factor $\frac{\alpha }{{\tilde V}}$ goes from purely imaginary to partly real (dashed and dotted lines: Δ_ac_ set to ½ resp. 1/8th the antenna linewidth).

While the absolute real frequencies of the two perturbed QNMs are not useful as information due to the disk size disorder, their difference is, since the random variations due to disk diameter disorder cancel out. Figure 6a, b reports the systematic mode separation (blue) and the difference in linewidth (red) for the symmetric and asymmetric QNM for the hybridization of antenna dimers with two different cavity modes, namely, the *m* = 86 mode of the first radial order (case of Fig. 5) and a mode with substantially different azimuthal quantum numbers *m* = 80 and mode volumes (second radial order). The period of the oscillation in Δ*θ* fitted from the experimental data is commensurate with the antinode spacing of the QNMs set by *m*, while the magnitude of the perturbation is markedly smaller for QNMs of radial order 2, commensurate with the larger mode volume. We traced similar results for all the WGM modes within the bandwidth of our laser, which amounts to 4 combinations of azimuthal and radial quantum numbers in total. Panel 6e summarizes the match between the azimuthal quantum number extracted from bare cavity spectroscopy (horizontal axis) and the value extracted from fitting Eq. (4) to the measured traces of linewidth versus antenna spacing.

Of the striking features predicted by our analysis, the experiments in Figs. 5 and 6a, b directly confirm that (I) the magnitude of the mode splittings varies inversely with the cavity mode volume and (II) the periodicity of the splitting with antenna separation varies with the azimuthal mode number as cos(*m*Δ*θ*). As this experiment was conducted with a fixed antenna size (varying the separation *d* as well as *m* and *V* in Eq. (4) but not *α*), it did not give access to two other salient predictions, namely, that (III) the tradeoff between real and imaginary contributions to the mode splitting depends on the phase of the polarizability *α* and that (IV) at very small separations, near-field hybridization should change the detuning behaviour. Regarding the phase of the polarizability, for Figs. 5 and 6a, b, the antenna size was such that the antennas were close in resonance to the cavities. Since within less than a linewidth from the plasmon resonance antenna polarizability is almost fully imaginary, the cavity perturbation should be almost entirely through the cavity damping rate and not through a real frequency shift. Indeed, Fig. 6a, b directly shows that for these structures, the splitting in frequency (real part) is much smaller than the maximum difference in linewidth. In a second experiment (Fig. 6, panel c for the real and imaginary part of the difference frequency between symmetric and asymmetric QNMs and panel d showing mode linewidths), we also studied a family of devices with deliberately smaller, i.e., blue-detuned, antennas, further focusing on a range of small antenna separations. The results of these samples highlight the role of the phase of the polarizability. For the chosen detuning Δ_*ac*_ = *ω*_*c*_−*ω*_*a*_ of approximately half the antenna linewidth *γ*_*a*_ = *γ*_*i*_ + *γ*_rad_, the polarizability had an approximately *π*/4 phase, as opposed to the *π*/2 on resonance. Consistent with the regime Re(*α*) ≈ Im(*α*), the mode splitting in the real and imaginary parts of the frequency (representing the resonance frequency and half damping rate, respectively) are now approximately equal in size. Overall, the splittings are smaller, however, owing to the fact that the polarizability of the antennas is off-resonant at the cavity frequency. In fact, we argue that one can *use* the ratio of the real and imaginary mode splittings to estimate the ratio of *Re*(*α*) to *Im*(*α*) through Eq. (4), while one can estimate the magnitude of *α* by comparing the overall size of the complex-frequency shift with the cavity mode volume. Figure 6f illustrates this idea. For all data sets, we extracted the complex prefactor $\alpha /\tilde V$ in Eq. (4). The magnitude is taken from the amplitude of the cosine behaviour for the absolute value of the complex-frequency shift, while the phase of $\alpha /\tilde V$ is taken from the complex argument of ${\mathrm{\Delta }}\tilde \omega = {\mathrm{\Delta }}\omega + i\frac{{{\mathrm{\Delta }}\gamma }}{2}$, where we have averaged over all datapoints with Δ*θ* > 2.5° to avoid the regime of near-field hybridization discussed below. The data sets with the antennas resonant near the interrogation frequency, i.e., near 780 nm, result in $\alpha /\tilde V$ on the imaginary frequency axis. This is commensurate with the notion that the QNM mode volume $\tilde V$ is essentially real for high-Q cavities, while the on-resonance polarizability of a plasmon antenna is imaginary. The data further clearly show the effect of the mode volume (*n*_*r*_ = 2 radial order mode appears at significantly smaller $\alpha /\tilde V$ for the same antenna size, i.e., fixed *α*). If one would be able to tune through the resonance of an antenna, one would expect $\alpha /\tilde V$ to sweep out a circle in the complex plane. The data sets with smaller antennas (Fig. 6f, lower-left datapoints) are indeed distinctly shifted in phase by approximately *π*/4, equivalent to a detuning by approximately half the antenna linewidth [antenna resonance near 630 nm].

Finally, our theoretical analysis projected that at very small antenna separations, deviations from the simple oscillatory dependence of mode splitting on the scatterer separation would set in. While only a few devices in our sample set access this regime, Fig. 6c, d indeed reveals that for the smallest antenna separations, the cavity perturbation does not follow the simple oscillatory dependence of mode splitting with the antenna separation. Instead, at the smallest separations, the system response is dominated by a very strong broadening of the antisymmetric cavity mode. This observation is a manifestation of the near-field hybridization of the two antennas.

## Discussion

We have reported a simple model for the emission enhancement properties of multimode, multi-antenna hybrid plasmon-photonic resonators, in particular focusing on WGM cavities coupled to plasmon antenna dimers. The model projects that these hybrids sustain similarly high hybrid Purcell factors as hybrids with only a single plasmon antenna but with the added benefit that one can tailor where the emission goes, with the freedom to arrange for branching ratios anywhere between symmetric and unidirectional circulation. As an example, if one would make a side-coupled waveguide the dominant loss channel for the cavity, then one could selectively extract light from emitters located at one of the antennas from just one waveguide port. Conversely, balancing the phase and amplitude of the two-waveguide-input port would enable the selective excitation of emitters placed at just one of the antennas or the other. Our analysis shows that the perturbative effects of the antennas are tailorable through the phase relation set by antenna placement, which in turn controls the interferences required for unidirectionality and linewidth/lineshift control. The essential physics of this hybridization is confirmed by experiments in an experimental platform based on silicon nitride microdisks and aluminium nano-antennas. While most systematic cavity perturbation experiments to date had to resort to scanning probe microscopy or scanning to avoid having to compare different devices with their inevitable spread in fabricated dimensions, our experimental platform is sufficiently reproducible to systematically *compare* plasmon-antenna-induced perturbations between devices, even for those narrowband cavities having GHz linewidths. The observations confirm our model for the hybridization physics, suggesting that high-Q plasmonic hybrid modes indeed offer an advantageous LDOS and a unidirectional light-matter coupling. The next step will be to actually demonstrate this unidirectional light-matter coupling. This would require a localized placement of emitters, such as quantum dots or fluorophores, at one of the antennas. The directionality of emission can already be demonstrated with ensembles of emitters packed in volumes of approximately 100 × 50 × 50 nm^3^ enclosing the antenna, as could be obtained by electron beam lithography of polymer resist doped with fluorophores. For the vision of hybrid nanophotonics for quantum optics on a chip, the challenge would rather be to locate a *single* quantum emitter. This highly challenging placement may be possible by extending the multi-step lithography approach to emitter placement reported by Curto et al.^27^, provided that a functionalization recipe to attach quantum dots to aluminium is available or alternatively could be possible with scanning probe microscopy using a fluorescent tip. A main challenge will lie in the fact that the unidirectionality occurs over a bandwidth equal to the linewidth of the hybrid modes (<0.1 nm, up to 1 nm possible with antenna-disk hybrids), while room-temperature emitters have a far larger linewidth (20 nm for typical quantum dots)^39^. We furthermore note that multimode antenna-cavity hybrids can also be of interest for controlling ensembles of emitters (distributed or localized), e.g., in the context of directional lasing, as has been already discussed for WGM cavities in the context of PT-symmetry and exceptional points in ref. ^41^. To this end, it would be necessary to enrich the system with a broken symmetry, for instance, by introducing a third antenna or considering unequal placement in the radial mode profile^29,40^. Further degrees of freedom for engineering unidirectionality would be offered by using polarization degrees of freedom. While we have considered only radially polarizable electric dipole antennas in this work, in fact, high-index dielectric particles and metallic split-ring shapes can show electric, magnetic, and coupled magneto-electric dipole polarizabilities, as reported in recent works on Janus, Kerker, and Huygens scatterers^42,43^. The excitation of suitable linear combinations of these moments is intrinsically associated with directional scattering. The microdisk geometry has near fields very similar to the non-transverse field of waveguides that have recently been used to generate circular dipoles in scatterers, with concomitant path-to-helicity conversion^44,45^. This provides a route to connect antenna-disk systems to chirality/helicity-specific light-matter interactions that also include emitters with magnetic dipole character^46^. Our QNM theoretical framework could be extended to address tensorial and magneto-electric response functions of scatterers or emitters. Finally, we note that multimode antenna-cavity hybrids will also offer new opportunities for high-Q particle sensing^19^, with all the benefits of plasmonic hotspots, as well as for exploring novel regimes of interaction in molecular optomechanic proposals^8^.

## Materials and methods

### QNM approach

For a set of *i* *=* *1… M* antennas of polarizability ***α***_*i*_, the induced dipole moments are given by^23,35^

5 $${\mathbf{p}}_i = {\it{\epsilon }}_0{\boldsymbol{\alpha}} _i[{\mathbf{E}}_{{\mathrm{dr}},{\mathrm{i}}} + {\mathbf{E}}_{{\mathrm{bs}},{\mathrm{i}}} + {\mathbf{E}}_{{\mathrm{dp}},{\mathrm{i}}}]$$

where ***α***_*i*_$(\omega )$ is the dipole polarizability tensor (normalized by vacuum permittivity *ε*_0_), ${\mathbf{E}}_{{\mathrm{dr}},{\mathrm{i}}}({\mathbf{r}}_i,\omega )$ represents an externally applied driving field, ${\mathbf{E}}_{{\mathrm{bs}},{\mathrm{i}}}({\mathbf{r}}_i,\omega )$ is the field radiated by the dipole *i* and scattered back onto *i* by the environment, and the term ${\mathbf{E}}_{{\mathrm{dp}},{\mathrm{i}}}({\mathbf{r}}_i,\omega )$ quantifies the field exerted on dipole *i* due to fields scattered by all dipoles labelled by *j* ≠ *i*. In the hypothesis where all polarizable objects are immersed in the same isotropic medium of refractive index *n*, one can decompose the system dyadic Green’s function ${\mathbf{G}}({\mathbf{r}},{\mathbf{r}}\prime ,\omega )$ such that **G** = **G**_0_ + Δ**G**, where ${\mathbf{G}}_0({\mathbf{r}},{\mathbf{r}}\prime ,\omega )$ represents the homogeneous Green’s function of a medium of index *n* and ${\mathrm{\Delta }}{\mathbf{G}}({\mathbf{r}},{\mathbf{r}}\prime ,\omega )$ is referred to as the Green’s function of the scattered field. With these definitions, we can formally express

$${\mathbf{E}}_{{\mathrm{dp}},{\mathrm{i}}} = \mathop {\sum }\limits_{j \ne i} \mu _0\omega ^2{\mathbf{G}}({\mathbf{r}}_i,{\mathbf{r}}_j,\omega ){\mathbf{p}}_j$$

and

$${\mathbf{E}}_{{\mathrm{bs}},{\mathrm{i}}} = \mu _0\omega ^2{\mathrm{\Delta }}{\mathbf{G}}({\mathbf{r}}_i,{\mathbf{r}}_i,\omega ){\mathbf{p}}_i$$

where *μ*_0_ is the vacuum permeability. We then use a partial QNM expansion of Green’s function^23,36^

6 $${\mathrm{\Delta }}{\mathbf{G}} = \frac{1}{{\mu _0\omega ^2}}\mathop {\sum }\limits_{k = 1}^N \frac{{ - \tilde \omega _k}}{{\omega - \tilde \omega _k}}{\tilde{\mathbf E}}_k({\mathbf{r}}) \otimes {\tilde{\mathbf E}}_k({\mathbf{r}}\prime ) + \delta {\mathbf{G}}_N$$

where ${\tilde{\mathbf E}}_k({\mathbf{r}})$ is the normalized electric field of the QNM indexed by *k*, $\tilde \omega _k = \omega _k + i\frac{{\gamma _k}}{2}$ is its complex frequency (*ω*_*k*_ and *γ*_*k*_ being the resonance frequency and linewidth, respectively), and $\delta {\mathbf{G}}_N({\mathbf{r}},{\mathbf{r}}\prime ,\omega )$ is the residue of the decomposition, accounting for all other modes of the system besides the ones explicit in the sum and non-resonant terms. This starting point is similar to a previous work^33^, where we examined perturbation theory for the interaction between a single polarizable object and a cavity mode, adapted to address multiple perturbers and cavity modes. In this work, we further assume that two initially degenerate cavity modes are dominant (*N* = 2), as appropriate for a WGM cavity, and that all other QNMs and non-resonant interactions grouped in *δ***G**_*N*_ can simply be neglected. These non-resonant interactions would be tedious to calculate accurately in a real geometry;^37^ however, one would expect them to be dominated by an electrostatic/near-field 1/*r*^3^ term interaction that is captured in **G**_0_.

We focus on the specific case of antennas interacting with a single degenerate pair of WGMs, as would be the case in a microdisk, microtoroid or microsphere cavity (as in Fig. 1). This implies the specific choice *N* = 2 and a pair of symmetric (s) and antisymmetric (as) mode functions of the form (in cylindrical coordinates (*r*, *θ, z*))

7 $${\tilde{\mathbf E}}_s \cdot {\mathbf{e}}_r = {\mathrm{cos}}(m\theta )\tilde E_r(r,z)$$

8 $${\tilde{\mathbf E}}_{as} \cdot {\mathbf{e}}_r = {\mathrm{sin}}(m\theta )\tilde E_r(r,z)$$

Note that from these normalized QNMs, clockwise and anticlockwise combinations can be formed through ${\tilde{\mathbf E}}_{cw/ccw} = \frac{1}{{\sqrt 2 }}({\tilde{\mathbf E}}_s \pm i{\tilde{\mathbf E}}_{as})$. The following considers antennas that are polarizable only along their long axis by TE WGMs (relevant for nanorods near resonance, aligned along the radial direction of a microdisk) and that are offset in the azimuthal direction (angle *θ*) but with a fixed radial position *r* on the edge of the microring cavity (see Fig. 1). Under these assumptions, the only relevant functional dependence on the antenna position is through *θ*_*i*_ (angle parametrizing the nanorod location) or, equivalently, the antenna angular separation Δ*θ* *=* *θ*_2_ − *θ*_1_, while the QNM strength at the antenna position (*r*_*i*_,*z*_*i*_) (resp. distance of antenna to the origin, height of the antennas relative to the disk plane) is set by $\tilde E_r(r_i,z_i)$, which is directly related to the on-resonance local density of states (LDOS) enhancement at the location of the antenna.

We now consider the emission enhancement of a dipole emitter placed in the vicinity of one of the nano-antennas. Therefore, we calculate the LDOS enhancement, which is defined as the total work required to maintain a drive dipole moment **p**_dr_ located at **r**_dr_:

9 $$P = \frac{\omega }{2}Im({\mathbf{p}}_{{\mathrm{dr}}}^ \ast \cdot {\mathbf{E}}_{{\mathrm{tot}}}({\mathbf{r}}_{{\mathrm{dr}}},\omega ))$$

normalized to the power required to drive the same dipole in free space given by Larmor’s formula $P_0 = \frac{{\omega ^4\parallel {\mathbf{p}}_{{\mathrm{dr}}}\parallel ^2}}{{12\pi {\it{\epsilon }}_b{\it{\epsilon }}_0c^3}}$, where *ω* is the driving frequency, **E**_tot_ is the total field radiated by the dipole evaluated in the presence of the cavity, and *ε*_*b*_ is the permittivity of the homogeneous isotropic, non-absorptive background medium. To obtain the relevant quantities, we substitute as the drive field **E**_dr,i_ in Eq. (1) the field imposed by a drive dipole, that is,

$${\mathbf{E}}_{{\mathrm{dr}},{\mathrm{i}}} = \mu _0\omega ^2{\mathbf{G}}({\mathbf{r}}_i,{\mathbf{r}}_{{\mathrm{dr}}},\omega ){\mathbf{p}}_{{\mathrm{dr}}}$$

solve for the induced (antenna) dipoles **p**_*i*_, and then calculate the total field returning to the drive dipole as

10 $${\mathbf{E}}_{{\mathrm{tot}}} = \mu _0\omega ^2\left[{\mathbf{G}}({\mathbf{r}}_{{\mathrm{dr}}},{\mathbf{r}}_{{\mathrm{dr}}},\omega ){\mathbf{p}}_{{\mathrm{dr}}} + \mathop {\sum }\limits_{i = 1}^M {\mathbf{G}}({\mathbf{r}}_{{\mathrm{dr}}},{\mathbf{r}}_i,\omega ){\mathbf{p}}_i\right]$$

Finally, we also reconstruct the directionality of emission into the cavity, i.e., how emission into the cavity modes is distributed over the clockwise and anticlockwise directions. To this end, we exploit the fact that the excitation of the symmetric and asymmetric degenerate modes ($\tilde \omega _1 = \tilde \omega _2 \equiv \tilde \omega$) is given in the QNM formalism as^38^

11 $$a_{s,as} = \frac{{ - \tilde \omega }}{{\omega - \tilde \omega }}\left[{\tilde{\mathbf E}}_{s,as}({\mathbf{r}}_{{\mathrm{dr}}}) \cdot {\mathbf{p}}_{{\mathrm{dr}}} + \mathop {\sum }\limits_{i = 1}^M {\tilde{\mathbf E}}_{s,as}({\mathbf{r}}_i) \cdot {\mathbf{p}}_i\right]$$

Since the (anti)clockwise mode amplitudes are then proportional to $a_{cw/ccw}(\omega ) = \frac{1}{{\sqrt 2 }}(a_s(\omega ) \mp ia_{as}(\omega ))$, we can introduce the directivity parameter $\sigma = \frac{{|a_{cw}|^2 \,-\ |a_{ccw}|^2}}{{|a_{cw}|^2 \,+\, |a_{ccw}|^2}} = \frac{{|a_s \,-\, ia_{as}|^2 \,-\, |a_s \,+\, ia_{as}|^2}}{{|a_s \,-\, ia_{as}|^2 \,+\, |a_s \,+\, ia_{as}|^2}}$, which is (−)1 if all light in the cavity is circulating in the (anti)clockwise cavity mode or 0 if light is distributed equally over both circulation directions.

The complex-frequency analysis follows by taking Eq. (5) through Eq. (11) with no driving terms, which leads to

12 $${\mathbf{p}}_i = {\it{\epsilon }}_0{\boldsymbol\alpha}_i (\omega )\left[ {\mu _0\omega ^2\mathop {\sum }\limits_{j = 1}^M {\mathbf{G}}_0({\mathbf{r}}_i,{\mathbf{r}}_j,\omega ){\mathbf{p}}_j + \mathop {\sum }\limits_{k = s,as} a_k{\tilde{\mathbf E}}_k({\mathbf{r}}_i)} \right]$$

keeping in mind that we neglect the residue *δ***G**_*N*_ of the QNM expansion.

### Sample fabrication

We use a two-step lithography to realize Si_3_N_4_ disks, hybridized with aluminium antennas. First, we fabricate Si_3_N_4_ on pyramidal silicon pedestals by electron beam lithography and reactive ion etching from silicon wafers with a 200 nm LPCVD layer of Si_3_N_4_ (Lionix BV). After base piranha cleaning, we perform e-beam lithography in 450 nm of CSAR 62 resist (All Resist GmbH) at 50 keV (Raith Voyager), using a 0.4 nA current and 160 μC/cm^2^ dose. After development in pentyl-acetate followed by an o-xylene dip, the samples are post-baked at 130 °C for 1 min to harden the resist to act as a plasma etch mask. After plasma etching through the nitride (RIE-ICP, SF_6_/CHF_3_ chemistry), we remove the resist using acetone and a base piranha clean, immediately followed by a Si underetch (KOH) to create free-standing disk edges. To realize the antennas, we then spin-coat a MMA/PMMA bilayer resist stack (120/60 nm, as measured at the edge of the cavity) to perform a second e-beam step aligned to the first. After e-beam writing (500 μC/cm^2^ dose) and development in a 1:3 methyl isobutyl ketone and isopropanol mixture, we perform aluminium evaporation (thermal evaporator at a 0.05 nm/s evaporation rate, targeting a 40 nm thickness) and lift-off in acetone at 40 °C. Finally, we ensure that the samples are accessible to optical fibre taper coupling by using a diamond saw to remove a 150 μm thick layer from the entire sample, except for a 150 μm wide ridge on which the structures stand. During this process, the sample is covered by a protective polymer resist that is stripped after sawing (Microposit S1800). This results in 15 μm diameter and 200 nm thick Si_3_N_4_ microdisks with two ≈130 nm long, ≈50 nm wide and ≈40 nm thick radially oriented Al antennas, placed 300 nm from the disk edge. Two samples are made with slightly different electronic doses for the second lithographic step. This results in a length difference between the two samples sufficient to ensure that for one sample, the antennas are almost on resonance with the cavity (operation near a 780 nm wavelength), while for the other sample, they were designed to be resonant at 630 nm.

### Optical setup and analysis framework

We interrogate the structures using a tapered optical fibre setup, sketched in Fig. 4b. The fibre is pulled to an adiabatic taper from a Corning HI 780C fibre that is in single mode at our operation wavelength of approximately 780 nm using an automated motorized hydrogen flame setup. The fibre is precisely made to approach the cavities using a piezo-stage setup, and excitation light is coupled in from an external-cavity diode laser that is widely tuneable yet narrowband (New Focus Velocity). The frequency axis of our cavity transmission scans is calibrated against a Fabry-Perot reference cavity (finesse >150, free spectral range of 10 GHz). We simultaneously collect reflected and transmitted signals on the photodiodes as well as scattered light (Fig. 4b). The scattered light is collected from the air side using a microscope objective and directed to a Basler CMOS camera, where we have access to both real space and k-space (angle-resolved) images. We can interrogate only modes of the first and second radial order because the fundamental mode is too strongly perturbed and therefore broadened by the antennas to be probed by tapered-fibre coupling and narrowband laser frequency scanning. The radial order mainly affects the cavity mode volume, as QNMs of increasing radial order have lower field amplitudes at the antenna locations.

To extract QNM frequencies and Q values from the raw data, we simultaneously fit reflection, transmission (data plotted in Fig. 5d), and scattering spectra taken from the radiation pattern. To this end, we perform a simultaneous fit to the Fano-like reflection, transmission and scattering data with a sum of 2 complex Lorentzians:

13 $$T(\omega ) = \left|1 - \frac{{\beta _{T;s}}}{{\omega - \tilde \omega _s}} - \frac{{\beta _{T;as}}}{{\omega - \tilde \omega _{as}}}\right|^2$$

14 $$R(\omega ) = \left|\frac{{\beta _{R;s}}}{{\omega - \tilde \omega _s}} + \frac{{\beta _{R;as}}}{{\omega - \tilde \omega _{as}}}\right|^2$$

15 $$S({\mathbf{k}},\omega ) = \left|\frac{{\beta _{S;s}({\mathbf{k}})}}{{\omega - \tilde \omega _s}} + \frac{{\beta _{S;as}({\mathbf{k}})}}{{\omega - \tilde \omega _{as}}}\right|^2$$

where the complex frequencies $\tilde \omega _{s,as}$ are common to all three fit functions, while the coefficients *β* are observable dependent. For scattering, we found that for a good fit, it is not necessary to determine the full-wave-vector-dependent yet frequency-independent amplitude functions $\beta _{S;s,as}({\mathbf{k}})$ for each QNM. Instead, for just obtaining the complex frequencies, taking only two wave vectors in the radiation pattern with a distinct Fano spectrum suffices (data plotted in Fig. 5e).

### Simulations

As input for our analytical model, we use an antenna polarizability that is taken as a Lorentzian polarizability with resonance frequency $\omega _0/(2\pi ) = 460$ THz and an Ohmic damping rate $\gamma _i/(2\pi ) = 19.9$ THz of gold, equivalent to taking the polarizability of a sphere and assuming a Drude model. We take a scatterer volume of 80 nm^3^ and incorporate radiation damping exactly as in ref. ^4^. This is equivalent to an on-resonance extinction cross section of 0.18 μm^2^ and scattering albedo of 85%, as typically achieved by large plasmonic dipolar antennas, and is close to the polarizability retrieved from full-wave simulations (ref. 4). We perform numerical calculations with a MATLAB® implementation of the theoretical model. COMSOL 5.2 finite element simulations are used to predict the mode structure of the fabricated microdisks and antennas.
